# 2SLGBTQ+ patients’ experiences in the pharmacy in British Columbia, Canada

**DOI:** 10.1177/17151635251360227

**Published:** 2025-09-12

**Authors:** Lillian Pei Chun Chen, Courtney Nicole Ng, Reema Mariam Abdoulrezzak, Mel Tsai, Sara Hiebert, Jasneek Kaur Manhas, Tristan Lai, Alex Tang

**Affiliations:** Faculty of Medicine, University of British Columbia, Vancouver, BC; Faculty of Pharmaceutical Sciences, University of British Columbia, Vancouver, BC; Faculty of Pharmaceutical Sciences, University of British Columbia, Vancouver, BC; Faculty of Pharmaceutical Sciences, University of British Columbia, Vancouver, BC; Faculty of Pharmaceutical Sciences, University of British Columbia, Vancouver, BC; Faculty of Pharmaceutical Sciences, University of British Columbia, Vancouver, BC; Faculty of Pharmaceutical Sciences, University of British Columbia, Vancouver, BC; Faculty of Pharmaceutical Sciences, University of British Columbia, Vancouver, BC; Faculty of Pharmaceutical Sciences, University of British Columbia, Vancouver, BC

## Abstract

**Background:**

Two-Spirit, lesbian, gay, bisexual, transgender, queer, intersex, and additional people who identify as part of sexual and gender diverse communities (2SLGBTQ+) experience worse health outcomes than cisgender heterosexual counterparts. This is attributable to structural oppression within the health care system, which manifests as health care providers’ lack of knowledge and training in 2SLGBTQ+ health.

**Methods:**

This cross-sectional, mixed-methods survey targeted 2SLGBTQ+ community members 14 and older in British Columbia to understand aspects of pharmacy care in the province. The survey asked participants about their experiences with the pharmacy environment, the pharmacist, and the competencies they thought were most important for pharmacists to provide inclusive care. The survey was disseminated online and live from October 24 to December 18, 2022.

**Results:**

After eligibility and exclusions, 195 records were included and analyzed. Some (46.2%) participants found the pharmacy environment inclusive. Half (51.8%) felt their needs as 2SLGBTQ+ people were met by the pharmacist. The most important pharmacist competencies identified were respect, knowledge of 2SLGBTQ+ health, and inclusive language. Participants also shared examples of their pharmacy experiences in free-text comments.

**Discussion:**

Despite increasing focus on equity, diversity, and inclusion in health care, there is still a significant gap in pharmacy care related to pharmacist competency, yet there is little progress in systematically training pharmacists on 2SLGBTQ+ competencies. Implementing 2SLGBTQ+ content into pharmacy programs is one approach to ensuring pharmacists are well-prepared to serve 2SLGBTQ+ communities.

**Conclusion:**

Findings highlighted 2SLGBTQ+ community-identified needs and served to inform curricular reform, professional development opportunities, and priorities of professional governing bodies as the pharmacy profession works towards more equitable health care for 2SLGBTQ+ communities. *Can Pharm J (Ott)* 2025;158:xx-xx.

**Knowledge into practice**

- Two-Spirit, lesbian, gay, bisexual, transgender, queer, intersex, and additional people who identify as part of sexual and gender diverse communities (2SLGBTQ+) in Canada face disproportionately worse health outcomes than cisgender heterosexual counterparts, because the health care system, pharmacists included, are unprepared to provide high-quality care for 2SLGBTQ+ communities.
- This study explored the largely unknown experiences of 2SLGBTQ+ patients in pharmacies in British Columbia and revealed that 2SLGBTQ+ patients’ needs were not well met and that the most important competencies pharmacists needed to provide inclusive care were respect, knowledge of 2SLGBTQ+ health, and inclusive language.
- Despite increasing efforts into prioritizing equity, diversity, and inclusion in health care, significant gaps remain in 2SLGBTQ+ care in British Columbian pharmacies, and intentional focus on education, training, and professional development reforms are needed to bridge these gaps.

**Mise En Pratique Des Connaissances**

- Les personnes bispirituelles, lesbiennes, homosexuelles, bisexuelles, transgenres, queer, intersexuées et d’autres personnes qui s’identifient comme faisant partie des communautés sexuelles et de genre diverses (2ELGBTQ+) au Canada sont confrontées à des résultats de santé disproportionnellement plus mauvais que leurs homologues cisgenres hétérosexuels, car le système de santé, y compris les pharmaciens, n’est pas préparé à fournir des soins de haute qualité aux communautés 2ELGBTQ+.
- Cette étude explore les expériences largement méconnues des patients 2ELGBTQ+ dans les pharmacies de la Colombie-Britannique et révèle que les besoins de ces patients ne sont pas bien satisfaits, et que les compétences les plus importantes dont les pharmaciens ont besoin pour fournir des soins inclusifs sont le respect, les connaissances sur la santé des personnes 2ELGBTQ+ et un langage inclusif.
- Malgré les efforts croissants déployés pour prioriser l’équité, la diversité et l’inclusion dans les soins de santé, des lacunes importantes subsistent dans les soins prodigués aux personnes 2ELGBTQ+ dans les pharmacies de la Colombie-Britannique. Il est donc nécessaire de mettre l’accent sur l’éducation, la formation et les réformes en matière de perfectionnement professionnel afin de combler ces lacunes.

## Background

It is well-known in literature that Two-Spirit, lesbian, gay, bisexual, transgender, queer, intersex, and additional people who identify as part of sexual and gender diverse communities (2SLGBTQ+) in Canada experience disproportionately worse health outcomes when compared to cisgender heterosexual counterparts.^1-3^ Studies show that 2SLGBTQ+ populations are at risk of worse mental health and higher rates of suicidality, chronic illnesses, cancer, sexually transmitted infections, and substance use disorders than the general population.^1,2,4,5^ These disparities can be attributed to the historical and persistent structural oppression of 2SLGBTQ+ communities, including alienation within the health care system.^6^ The systemic deprioritization of 2SLGBTQ+ communities’ health leads to poor access to health care and health care providers who lack the necessary skills to provide inclusive care.^6-8^

Pharmacists are the most accessible health care providers who are uniquely positioned to better serve 2SLGBTQ+ patients,^9,10^ but there are significant gaps in the current quality of pharmacy care. As evidenced in one study done in the United States, 41.6% of transgender and gender nonconforming patients worried about experiencing discrimination in pharmacies, and 13.3% avoided pharmacies because of previous embarrassing experiences.^11^ Additionally, studies based in the United States found that student pharmacists and pharmacists did not feel adequately trained to provide care to 2SLGBTQ+ communities.^12^ The limited research on the quality of 2SLGBTQ+ pharmacy care in Canada paints a dismal picture where pharmacists lack the knowledge required for inclusive care.^13^ One study showed that Two-Spirit individuals experienced racism, homophobia, and transphobia in the community pharmacy that resulted in avoidance of care.^14^ Literature focused on experiences in British Columbia (BC) is scarce, and a better understanding of current 2SLGBTQ+ care needs in BC is crucial for efforts to effectively improve 2SLGBTQ+ care.

This survey was conducted as part of the Promoting 2SLGBTQ+ Inclusion, Diversity, and Equity in Pharmacy Education (PrideRx) initiative, which sought to embed 2SLGBTQ+ competencies into the entry-to-practice Doctor of Pharmacy (PharmD) curriculum at the University of British Columbia (UBC). The findings from this survey informed curricular changes in the PharmD program with the aim of improving care for 2SLGTQ+ patients.

### Objectives

The objectives of this survey were (1) to understand the experiences of 2SLGBTQ+ communities when accessing pharmacy and pharmacist services in BC and (2) to identify key priorities when developing a 2SLGBTQ+ inclusive curriculum.

## Methods

This cross-sectional, mixed-methods study was conducted in BC, targeting 2SLGBTQ+ community members age 14 or older. Prior to dissemination, the study was piloted with 4 2SLGBTQ+ community members identified through partner community organizations. The survey contained 2 eligibility questions and 8 demographic questions followed by a series of 5-point Likert scale statements: 4 about the environment of the participant’s their pharmacy and 4 about their pharmacist. Open-ended questions allowed participants to elaborate on their pharmacy’s environment and their experiences with their pharmacist. The final portion of the survey asked participants to rank 8 areas of knowledge or skills most important for a pharmacist to provide inclusive care. Two optional open-ended questions at the end of the survey asked participants to list topics they felt were missing and provide further comments. The full survey is available in Appendix 1 (available online under Supplementary Materials).

Convenience and snowball sampling were used for this survey, which was disseminated via social media platforms of several local 2SLGBTQ+ community organizations, by word of mouth, and through the UBC Faculty of Pharmaceutical Sciences’ social media platforms. The survey was live from October 24 to December 18, 2022.

All responses were anonymous. Participants could skip any question except eligibility questions. Participants were eligible to enter a gift card draw as compensation for their time.

This study was funded by the Internal Seed Grant for Educational Leadership, from the Faculty of Pharmaceutical Sciences at UBC. This study received approval from the Behavioural Research Ethics Board of UBC (H22-01983) on October 19, 2022. Participants gave implied consent by completing the survey.

### Inclusion and exclusion criteria

Inclusion criteria for survey participation required participants to be at least 14 years old, residing in BC, and identifying as part of the 2SLGBTQ+ communities.

Each survey response was independently reviewed by 2 reviewers to determine whether the exclusion criteria were met. Survey responses were excluded if they were less than 75% complete, were duplicated, were non-English, or were likely non-human or bot-like in nature. Bot-like responses were defined as duplicate responses entered within a small-time frame (e.g., ≥10 responses in 30 seconds) or completed in less than 100 seconds, irrelevant or incoherent answers to free-text questions, or free-text fields containing text plagiarized from existing literature. Discrepancies between reviewers were resolved through discussion and consensus.

### Data analysis

Likert scale items and ranking data were analyzed descriptively. A mean rank and mode rank were calculated for each topic in the ranking question, and the distributions of ranks for each topic were represented as bar graphs. A pairwise comparison was conducted between the 3 topics with the highest mean rank to determine which topics were most frequently ranked higher than the other popular topics.^15^

Open-ended responses were analyzed qualitatively via content analysis. Two reviewers independently analyzed the qualitative data, and a third reviewer resolved discrepancies. Themes were reported based on frequency.

## Results

At the end of the 8-week period, 1720 responses were recorded. In the first step of exclusion, 162 responses were excluded for being incomplete, 91 were excluded for participants not belonging in the 2SLGBTQ+ communities, and 39 were excluded for respondents not being 14 or older residing in BC. Some responses were omitted for more than 1 reason. Then, responses were excluded for being bot-like, duplicated, or completed too quickly. Finally, 195 records were included.

### Demographics

Most participants were 26 to 45 (42.1%) and 20 to 25 (41.0%) years old, and the majority (54.4%) identified as Black, Indigenous, or People of Colour (BIPOC). Of the sample, 28% identified as intersex, 61% identified as transgender, and 30% identified as Two-Spirit or Indigiqueer. Table 1 contains a further breakdown of demographics.

**Table 1:** Demographics of included participants, *N* = 195

	*n*	%
Age		
14-19 y	20	10.3
20-25 y	80	41.0
26-45 y	82	42.1
46-65 y	10	5.1
≥65 y	2	1.0
Prefer not to answer	1	0.5
Race/ethnicity		
Black, Indigenous, or Person of Colour (BIPOC)	106	54.4
Not BIPOC	89	45.6
Two-Spirit or Indigiqueer		
Yes	59	30.3
No	47	24.1
Blank*	89	45.6
Intersex		
Yes	55	28.2
No	140	71.8
Transgender		
No	76	39.0
Yes	119	61.0
Gender identities†		
Agender	22	11.3
Cisgender man	38	19.5
Cisgender woman	29	14.9
Genderfluid	26	13.3
Non-binary	52	26.7
Questioning	14	7.2
Trans-man	37	19.0
Trans-woman	34	17.4
Transfeminine	16	8.2
Transmasculine	22	11.3
Self-describe	2	1.0
Sexual identities†		
Asexual	4	2.1
Bisexual	74	37.9
Demisexual	22	11.3
Gay	52	26.7
Lesbian	30	15.4
Pansexual	33	16.9
Queer	68	34.9
Questioning	10	5.1
Straight/heterosexual	15	7.7
Self-describe	5	2.6

* Participants were shown the question asking whether they are Two-Spirit or Indigiqueer only if they answered “yes” to being BIPOC.

† For these categories, participants could select multiple options.

### Pharmacy environment

Participants’ evaluation of their pharmacies’ environment is reported in Table 2. Many participants (46.2%) found the physical environment of their pharmacy inclusive, and 52.3% found an adequate selection of products for their sexual and health needs. Only 36.5% found the washrooms inclusive of all genders, and often (17.5%), participants could not evaluate the inclusivity of washrooms, possibly because they were unavailable. The majority (69.7%) felt it was important to be able to speak with a 2SLGBTQ+ staff member. In open-ended answers, 20.0% of participants identified the presence of 2SLGBTQ+ signage and health pamphlets in their pharmacy as signs of an inclusive space. Participants shared that inclusive displays made them feel “immediately at ease” and signalled that it was safe to “share information about their identity”. Other features of inclusive pharmacies included the presence of queer-inclusive products, such as binding and tucking products, and inclusive forms and documentation. Table A1 (Appendix 2, available online under Supplementary Materials) summarizes qualitative data on inclusive aspects of pharmacy environments.

**Table 2:** Participants’ Likert responses evaluating the pharmacy environment (*N* = 195)

Statement	Strongly disagree	Somewhat disagree	Neither agree nor disagree	Somewhat agree	Strongly agree	Not applicable or blank
I feel like the pharmacy washrooms are inclusive of all genders.	28 (14.4)	28 (14.4)	34 (17.4)	43 (22.1)	28 (14.4)	34 (17.5)
I feel like there is an adequate selection of shelf products for my sexual and health care needs.	14 (7.2)	44 (22.6)	29 (14.9)	58 (29.7)	44 (22.6)	6 (3.0)
I feel that the physical environment of the pharmacy is 2SLGBTQ+ inclusive.	14 (7.2)	27 (13.8)	60 (30.8)	59 (30.3)	31 (15.9)	4 (2.0)
I feel that it is important that there is a 2SLGBTQ+ pharmacy staff member whom I can speak to.	7 (3.6)	10 (5.1)	37 (19.0)	49 (25.1)	87 (44.6)	5 (2.5)

Data are expressed as *n* (%). 2SLGBTQ+, Two-Spirit, lesbian, gay, bisexual, transgender, queer, intersex, and additional people who identify as part of sexual and gender diverse communities.

Some participants reported negative experiences, the most common being the lack of private spaces for pharmacist consultation (11.2%). Some noted that the products available at a pharmacy and the messaging associated can reflect a non-inclusive environment (10.4%); for example, gendered product or aisle labels like “feminine hygiene products” uphold cisheteronormativity. Table A2 in Appendix 2 summarizes qualitative findings on non-inclusive aspects of pharmacy environments.

While pharmacy environments were important, participants also emphasized pharmacy professionals’ role in creating an inclusive space, with 1 participant noting that “[staff] actions and words [are what promotes] inclusivity”. Having open-minded and respectful staff (19.2%) was the second most commonly identified factor contributing to inclusive pharmacies. Staff who were knowledgeable and trained to use inclusive language were also important facilitators for inclusivity.

### Experience with the pharmacist

When speaking with a pharmacist, most participants reported positive experiences. Most were often or always comfortable sharing their identities with pharmacists (56.9%) and correcting pharmacists about their identities (56.4%). Over half (51.8%) of participants felt that their needs as 2SLGBTQ+ people were met. When asked if they felt respected when speaking to a pharmacist, 49.7% responded affirmatively, while 27.7% were ambivalent. Likert responses are summarized in Table 3. In open-ended answers, 42.2% of responses identified clinical competence as an important quality that determined whether pharmacists could meet their needs. Clinical competence included providing participants with their prescriptions and providing appropriate medication information without making uncomfortable assumptions. Another frequently identified quality of pharmacists that helped meet 2SLGBTQ+ needs was respect (40.7%). Conversely, the most cited reasons for a pharmacist failing to meet the needs of 2SLGBTQ+ participants were the lack of competence in 2SLGBTQ+ health (46.2%) and making heteronormative assumptions or asking intrusive questions (28.8%). Participants reported that pharmacists were not knowledgeable about transgender care or hormone use, and thus “trans people [were] forced to be experts in their own care”. Participants also reported pharmacists denying access to syringes required for hormone therapy and experiences having to “constantly fight . . . to validate [their] medication”. Participants also commonly reported being misgendered or deadnamed (25.0%) repeatedly despite previous corrections. Further details are reported in Tables A3 and A4, both in Appendix 2.

**Table 3:** Participants’ Likert responses evaluating their experiences with pharmacists (*N* = 195)

Statement	Strongly disagree	Somewhat disagree	Neither agree nor disagree	Somewhat agree	Strongly agree	Not applicable or blank
I feel respected as a 2SLGBTQ+ person.	5 (2.6)	28 (14.4)	54 (27.7)	49 (25.1)	48 (24.6)	11 (5.7)
I feel comfortable correcting my pharmacist about my gender or sexual identity.	24 (12.3)	32 (16.4)	15 (7.7)	64 (32.8)	47 (24.1)	13 (6.7)
I feel that my needs are met as a 2SLGBTQ+ person.	10 (5.1)	29 (14.9)	42 (21.5)	54 (27.7)	47 (24.1)	13 (6.7)
I feel comfortable sharing my gender and sexual identity if asked.	27 (13.8)	35 (17.9)	15 (7.7)	58 (29.7)	52 (26.7)	8 (4.1)

Data are expressed as *n* (%). 2SLGBTQ+, Two-Spirit, lesbian, gay, bisexual, transgender, queer, intersex, and additional people who identify as part of sexual and gender diverse communities.

### Pharmacist education

When asked to rank a list of topics of pharmacist education from the most important to the least important, with rank 1 being the most important, the highest ranked topics were, in order, “treat me respectfully without assuming or stereotyping” (“Respect”), “have knowledge about 2SLGBTQ+ health topics” (“Knowledge”), and “address me using pronouns and language I am comfortable with” (“Inclusive language”). The mean ranks were 3.08, 3.25, and 4.18, respectively (Table 4). The histogram distributions of ranks for respect and knowledge were both left-skewed, with the mode rank being rank 1 (Figure A1 in Appendix 3, available online under Supplementary Materials). The mode rank for inclusive language was rank 2. In pairwise comparisons, respect was more frequently ranked above knowledge and inclusive language than vice versa, which is consistent with the calculated mean rank (Table A5 in Appendix 2).

**Table 4:** Participants’ rankings of the important pharmacist skills that enable them to provide inclusive care

	Mean rank	Mode rank
Treat me respectfully without assuming or stereotyping.	3.08	1
Have knowledge about 2SLGBTQ+ health topics.	3.25	1
Address me using pronouns and language I am comfortable with.	4.18	2
Understand how social factors can affect the health of people in my communities.	4.50	6
Create an inclusive physical space that shows me I am accepted for who I am.	4.51	6
Listen to me and my experiences as a 2SLGBTQ+ person.	5.28	5, 7*
Recommend local 2SLGBTQ+ resources.	5.38	6
Know about the history of 2SLGBTQ+ communities and various sexual and gender identities.	5.83	8

The item ranked 1 was the most important, and 8 was the least important. A mean rank was calculated for each item. 2SLGBTQ+, Two-Spirit, lesbian, gay, bisexual, transgender, queer, intersex, and additional people who identify as part of sexual and gender diverse communities.

* There were 2 modes. Both were reported instead of reporting an average.

When asked to comment on what other skills participants would like pharmacists to have, the most common theme was respect, re-emphasized by 36.0% of responses despite already ranking high numerically. One participant said that respect was the “bare minimum”. Sensitivity to 2SLGBTQ+ experiences was also brought up by 26.0% of responses, a concept that encompasses considerations like providing privacy and ensuring confidentiality to not “out” 2SLGBTQ+ participants to others, understanding common challenges community members can face such as discrepant legal names and insurance documentation, and respecting the patients’ gender and sexual identities as an important but not all-encompassing part of their health care journey. Participants also wanted to see pharmacists take accountability for the inclusivity of the care provided (14.0%) and demonstrate commitment to inclusivity (16.0%).

One-third of responses noted that further knowledge-based training was required for pharmacists. Out of these responses, the most identified topic was intersectionality (47.1%; 8/17), with participants sharing that “class/socioeconomic status, age, disability, race/ethnicity, size [are] all relevant”. Knowledge in transgender health was also commonly mentioned (41.2%). See Table 5 for details.

**Table 5:** Participant-identified themes of topics that pharmacists need more education in (*N* = 17, using only responses that mentioned pharmacist competency)

Theme	%	*n*	Examples
Intersectionality	47.1	8	“I would like my pharmacist to have knowledge of intersectionality and treat queer Black Indigenous and people of colour with respect, and queer people with respect regardless of their background or financial status. Since some queer people get treated better if they are white, cis, attractive, financially well-off, etc., and queer resources are centred around the experience of white queers.”
Trans health	41.2	7	“[Pharmacists should] understand that someone assigned female at birth may just be a man, not a trans man or transmasculine, and the converse for someone assigned male at birth. Transition can just be an event with a beginning and end, not an identity of transgender. Someone who transitioned to male ages ago may not want to be identified as transgender.”
Sexual health	11.8	2	“I think all pharmacists should have knowledge on queer sexual/reproductive health as well specifically. Birth control might be important for helping prevent pregnancy but it can also be a vital part of gender-affirming care. Taking hormones could greatly impact the effects of many medications and vitamins. Many young queer people do not have parents to ask questions about sexual and reproductive health, and with doctor shortages around Canada, many queer people may be stuck with many important questions unanswered that can possibly be answered by pharmacists.”
Pharmacokinetic knowledge	5.9	1	“[Pharmacists should] have relevant training, 2SLGBTQ+ health needs, and physiological drug maintenance data.”
Aging	5.9	1	“[A pharmacist] needs knowledge in HIV medications and interactions, knowledge of aging and gay men’s health and sexuality.”

2SLGBTQ+, Two-Spirit, lesbian, gay, bisexual, transgender, queer, intersex, and additional people who identify as part of sexual and gender diverse communities.

## Discussion

Despite increasing focus on equity, diversity, and inclusion in health care, we found that over half of 2SLGBTQ+ individuals surveyed did not find their pharmacy’s environment inclusive. Participants identified gaps in the lack of private consultation spaces and gender-inclusive products. Participants also found care provided by pharmacists inadequate to meet their health care needs. These findings are consistent with literature^13,16^ and affirm the need for pharmacists to be clinically competent in all therapeutic areas, especially ones that are overrepresented in 2SLGBTQ+ patients.^10,17^ An Ontario-based study found that 43.9% of trans participants reported unmet health care needs, as compared to 10.7% in the cisgender population.^16^ The unmet needs can be attributed to a non-inclusive environment and lack of pharmacist competence, both of which were elucidated in this study and echoed in the literature. A study on 2SLGBTQ+ care provided by hospital-based pharmacists across Canada found that only 40.6% of participants indicated that their workplace promoted an affirming and inclusive environment through signage and visible displays, and less than one-third reported that they could confidently engage in conversations about gender-affirming hormone therapy and transgender cancer screening, which are 2 important aspects of 2SLGBTQ+ health.^13^ The research team was not aware of other studies comparing the importance of pharmacist competencies against one another; however, the 3 top competencies participants highlighted in our study, “respect”, “knowledge”, and “inclusive language”, were reflected in findings from numerous studies as facilitators of inclusive care.^6,18,19^ Pharmacists and pharmacies that are prepared for and competent in 2SLGBTQ+ care are critical facilitators in improving 2SLGBTQ+ health and can help patients feel better understood and more engaged in their own care.^20^

Findings from our study highlighted 2SLGBTQ+ patients’ pharmacy experiences and unmet needs in pharmacy education in BC, home to the third highest number of transgender and non-binary people among Canadian provinces and territories.^21^ In recent years, pharmacy professional organizations have shown increased support for 2SLGBTQ+ communities via messaging around 2SLGBTQ+ health promotion.^22-24^ The Canadian Council for Accreditation of Pharmacy Programs requires pharmacy programs across Canada to adequately develop students’ understanding of anti-oppression, including in the context of 2SLGBTQ+ care.^25^ Even with increased awareness of 2SLGBTQ+ needs at a regulatory level, little tangible change has occurred. The PrideRx initiative was the first organized effort in BC to create a longitudinal curriculum that trains future pharmacists to meet the health care needs of 2SLGBTQ+ communities. Efforts to introduce 2SLGBTQ+ content into the pharmacy curriculum are also few and far between in pharmacy programs across Canada, with the few programs taking on this task being trailblazers in the realm rather than the norm.^26-30^ It is clear that more work is needed to transform pharmacist training, and the community-identified priorities in pharmacist competencies can serve as a roadmap for current and future efforts to reform pharmacy curricula.

### Considerations for a 2SLGBTQ+ inclusive curriculum

Knowledge was ranked as the second most important pharmacist competency in this study and the most frequently named factor that determined whether pharmacists were meeting the needs of 2SLGBTQ+ communities. Our findings showed the importance of better education for current and future pharmacists to provide high-quality, inclusive, and dignified care to 2SLGBTQ+ communities, which requires demonstrating inclusivity in the physical environment, in communication, and in acknowledging the lived experiences of 2SLGBTQ+ patients. This multidimensional unmet need in the care of 2SLGBTQ+ communities suggests that an equally multidimensional approach is needed to equip health care providers with the necessary skills to provide inclusive care. Many skills and attitudes highlighted, like respect and sensitivity to 2SLGBTQ+ lived experiences, are not easily taught in single-instance, short learning activities. Exposure to different aspects of 2SLGBTQ+ health longitudinally throughout training and continuing education is necessary to build the skills, mindset, and the knowledge required to best serve this population and narrow the equity gap experienced by 2SLGBTQ+ communities. In response to these findings, the research team designed and implemented a longitudinal 2SLGBTQ+ curriculum in the UBC PharmD program. The top competencies, “respect”, “knowledge”, and “language”, were each introduced in program years 1 and 2 and then further reinforced and developed in years 3 and 4 (see Box 1). Participants identified topics of intersectionality and trans-health as important areas of knowledge, and these were taught through experiential practicums and dedicated lecture series, respectively. Future research on how to most effectively address the unmet educational needs identified in this study, whether it be through curriculum reform, continuing education programs, or professional development activities, is needed to tangibly improve the care of 2SLGBTQ+ communities.

Box 1 **Findings from this study highlighted the multidimensional skillsets pharmacists need to provide inclusive care. Key competencies identified were integrated longitudinally throughout the 4-year University of British Columbia PharmD program**

**Year 1 and year 2:** A mandatory lecture and workshop series introduced foundational knowledge of 2SLGBTQ+ health, inclusive language, and 2SLGBTQ+ lived experiences.

**Year 3 and year 4:** An elective course and a subsequent elective practicum built on the competencies developed in the earlier years. Knowledge gaps identified in this study were addressed through didactic lectures and experiential learning across years 3 and 4.

### Strengths and limitations

To the best of our knowledge, our team was the first to investigate 2SLGBTQ+ experiences in BC. This study fills an important gap in literature for this local context.

Another strength of this study was its ability to seamlessly translate knowledge into actionable change as part of a larger initiative to champion curricular change in UBC. The competency priorities identified directly informed course design.

Like other minoritized populations, 2SLGBTQ+ communities are known to be difficult to reach.^31^ In this study, participants were recruited through community-based organizations, which not only helped reach communities through a trusted source but served to build reciprocal relationships between the academic institution and community organizations.^31^ Although pragmatic and effective, this convenience sample may not accurately reflect the 2SLGBTQ+ communities in BC. Despite researchers intentionally reaching out to community-based organizations outside of Vancouver, fewer organizations outside of Vancouver engaged with survey dissemination, which likely resulted in an urban sampling bias.

It should also be noted that this study suffered from the presence of bots within the survey data. A gift card incentive was offered as an attempt to remunerate participants who shared their experiences; however, this potentially attracted fraudulent submissions. To protect the privacy of vulnerable participants, the research team opted not to track any identifying information, including IP addresses, and the complete anonymity of responses made it difficult to distinguish genuine and fraudulent responses. Despite the best efforts of the reviewers, genuine responses may have been removed from the study, and fraudulent responses may have been included, and ultimately, less than 10% of responses were retained for analysis. The presence of bots disproportionately affected the integrity of the quantitative data collected, as it was more difficult to detect bot activity in multiple choice answers versus free-text answers. As bots become more sophisticated, future studies should consider incorporating attention checks, open-ended questions, CAPTCHA tests, and asking the same question several different ways.^32,33^

The content validity of this survey was informed by team members’ lived experiences as members of 2SLGBTQ+ communities. Face validity was optimized through piloting the survey with community members and implementing survey mechanics like hover-over definitions for key terms to ensure participants understood the survey text.^34^ This survey was designed with patient experiences with outpatient pharmacy care in mind. This bias, reflected in the survey text, could lead to participants being less primed to share experiences from inpatient settings. Further studies of 2SLGBTQ+ experiences in other pharmacy settings will be valuable.

The research team recognizes that 2SLGBTQ+ identities are only a small part of participants’ lived experiences, and many participants also inhabit other intersectional identities that affect their experiences of pharmacy care. Subgroup analyses based on race and other intersectional identities were not pursued; however, this would be a relevant area of future study.

## Conclusions

This study focused on the experiences of 2SLGBTQ+ communities accessing pharmacy care in BC, and results were similar to studies done across Canada; more mindful work is required to improve the quality of 2SLGBTQ+ care in pharmacies. Our findings highlighted 2SLGBTQ+ community-identified needs and served to inform curricular reform, professional development opportunities, and priorities of professional governing and advocacy bodies as we work towards a more equitable health care system for 2SLGBTQ+ communities. ■

## Supplemental Material

sj-pdf-1-cph-10.1177_17151635251360227 – Supplemental material for 2SLGBTQ+ patients’ experiences in the pharmacy in British Columbia, Canada Supplemental material, sj-pdf-1-cph-10.1177_17151635251360227 for 2SLGBTQ+ patients’ experiences in the pharmacy in British Columbia, Canada by Lillian Pei Chun Chen, Courtney Nicole Ng, Reema Mariam Abdoulrezzak, Mel Tsai, Sara Hiebert, Jasneek Kaur Manhas, Tristan Lai and Alex Tang in Canadian Pharmacists Journal / Revue des Pharmaciens du Canada

sj-pdf-2-cph-10.1177_17151635251360227 – Supplemental material for 2SLGBTQ+ patients’ experiences in the pharmacy in British Columbia, Canada Supplemental material, sj-pdf-2-cph-10.1177_17151635251360227 for 2SLGBTQ+ patients’ experiences in the pharmacy in British Columbia, Canada by Lillian Pei Chun Chen, Courtney Nicole Ng, Reema Mariam Abdoulrezzak, Mel Tsai, Sara Hiebert, Jasneek Kaur Manhas, Tristan Lai and Alex Tang in Canadian Pharmacists Journal / Revue des Pharmaciens du Canada

sj-pdf-3-cph-10.1177_17151635251360227 – Supplemental material for 2SLGBTQ+ patients’ experiences in the pharmacy in British Columbia, Canada Supplemental material, sj-pdf-3-cph-10.1177_17151635251360227 for 2SLGBTQ+ patients’ experiences in the pharmacy in British Columbia, Canada by Lillian Pei Chun Chen, Courtney Nicole Ng, Reema Mariam Abdoulrezzak, Mel Tsai, Sara Hiebert, Jasneek Kaur Manhas, Tristan Lai and Alex Tang in Canadian Pharmacists Journal / Revue des Pharmaciens du Canada
