# Supplementary material for: 2SLGBTQ+ patients’ experiences in the pharmacy in British Columbia, Canada
Source: Can Pharm J (Ott). 2025 Sep 12;158(6):368–77. doi: 10.1177/17151635251360227 (PMC12432006; doi:10.1177/17151635251360227)
Supplement: sj-pdf-1-cph-10.1177_17151635251360227 – Supplemental material for 2SLGBTQ+ patients’ experiences in the pharmacy in British Columbia, Canada [file sj-pdf-1-cph-10.1177_17151635251360227.pdf]

APPENDIX 1

PRIDE-RX: Survey for Community Members

Start of Block: Block 2

Q15 **SURVEY FOR COMMUNITY MEMBERS AND PARTNERS**
You are invited to participate in this online survey because we want to learn about your pharmacy experiences as members of 2SLGBTQQIA+ communities in British Columbia. This urvey is open to 2SLGBTQQIA+ identifying residents of British Columbia who are 14 years old or older who have used pharmacy services.

Study Title
Promoting 2SLGBTQQIA+ Inclusion, Diversity, and Equity in Pharmacy Education (PRIDE-RX)

Principal Investigators

Alex Tang BSc(Pharm), RPh *(He/Him)*
Principal Investigator
Lecturer, Faculty of Pharmaceutical Sciences
The University of British Columbia
alex.tang@ubc.ca

Tristan Lai BSc(Pharm), RPh *(He/Him)*
Principal Investigator
Lecturer, Faculty of Pharmaceutical Sciences
The University of British Columbia
tristan.lai@ubc.ca

How long will this survey take?
5-10 minutes

What are the benefits to doing this survey?
Those who participate in this survey may opt into a random draw for a chance to win a $20 CAD e-gift card.

Is there any harm to doing this survey?
Some of the questions we ask in the survey may be sensitive. For example, we will ask about your gender and sexual identity and your previous experiences accessing pharmacy services, which may bring up unpleasant or distressing memories. Your participation is voluntary. You may exit the survey at any time without penalty. You are free to decline to answer any questions you do not wish to answer for any reason. Your decision to participate in this survey will not affect any service you may use or your place in the community you belong to.

Will the results from this survey be shared?
The results of this study may be published in academic journal articles or presented in conferences.

Will I be asked for my personal information?
This survey does not collect any identifying personal information such as your name, IP address, location, or contact information, therefore, your responses will be completely

anonymous. As the survey is anonymous, it will not be possible to withdraw the data once it has been submitted. No one will be able to identify you or your answers from this survey. You may optionally include your email address and name in a subsequent survey if you would like to be entered in a draw for a $20 CAD e-gift card. Please note that the contact information provided in the subsequent survey will not be linked to your survey response.

Who to contact?
If you have questions about this survey, how it is being conducted, or how your answers may be used, you may contact Tristan Lai (*he/him*) at tristan.lai@ubc.ca or Alex Tang (*he/him*) at alex.tang@ubc.ca. If you have any concerns or complaints about your rights as a research participant and/or your experiences while participating in this survey, please contact the Research Participant Complaint Line in the UBC Office of Research Ethics at 604-822-8598. You can also e-mail RSIL@ors.ubc.ca or use the toll-free number 1-877-822-8598.

Instructions
During the survey, you may encounter terms you are not familiar with. Hover over key terms to see the associated definitions. This survey and its features are optimized for a computer. While you may choose to respond on a mobile device, the hover over feature may not work.

Consent
By clicking the ‘Next’ button below, you agree that you have read the above information, understand it, and voluntarily agree to participate in this survey. You may decide to stop participating in this survey at any time by exiting this web page. If the survey is completed, it will be assumed that consent has been given.

This survey is funded by the UBC Scholarship of Teaching and Learning Internal Seed Grant Program for Educational Leadership. We would like to acknowledge that this research and the training of student pharmacists are being done at UBC Vancouver Campus, on the traditional, ancestral, and unceded territories of the xʷməθkʷəy̓əm (Musqueam) people. The Ethics ID number for this survey is H22-01983.

End of Block: Block 2

Start of Block: Screening Question

The 2SLGBTQQIA+ communities include folx who are Two-Spirit, lesbian, gay, bisexual, transgender, queer, questioning, intersex, asexual or identify as another gender or sexually diverse identity. **Do you identify as a member of the 2SLGBTQQIA+ communities?**

○ Yes *(Please note that this includes people who identify as "Questioning" and those whose identity is not explicitly captured in the 2SLGBTQQIA+ acronym.)* (1)

○ No (2)

End of Block: Screening Question

Start of Block: Block 8

Q34 Are you a resident in British Columbia aged 14 or older?

○ Yes (1)

○ No (2)

End of Block: Block 8

Start of Block: Gender and Sexual Identity

Q20 How old are you?

○ 14-19 (8)

○ 20-25 (1)

○ 26-45 (4)

○ 46-65 (5)

○ 65 or older (6)

○ Prefer not to answer (7)

Page Break

Q35 Do you self-identify as Black, Indigenous, or a Person of Colour (BIPOC)?

- ◯ Yes (1)
- ◯ No (2)

Display This Question:

If Do you self-identify as Black, Indigenous, or a Person of Colour (BIPOC)? = Yes

Q36 Do you identify as Two-spirit, ‘indigiqueer' or any analogous term?

- ◯ Yes (1)
- ◯ No (2)

Q37 Are you intersex?

- ◯ Yes (1)
- ◯ No (2)

Q38 Are you trans or transgender, or is your gender identity different from the one you were assigned at birth?

- ◯ Yes (1)
- ◯ No (2)

Display This Question:

If Are you trans or transgender A person whose gender identity differs from the sex they were assign... = No

Q41 What is your gender identity? (select all that apply)

- ▢ Agender A person who does not identify themselves as having a particular gender (1)
- ▢ Cisgender A person whose assigned sex at birth matches their gender identity man (2)
- ▢ Cisgender A person whose assigned sex at birth matches their gender identity woman (3)
- ▢ Genderfluid A person who does not identify as having a fixed gender (4)
- ▢ Non-Binary A person whose gender identity does not fall into the gender binary (i.e. male or female) (5)
- ▢ Questioning People who feel unsure about their gender identity or sexual orientation may identify with the term ‘questioning’ (6)
- ▢ If your identity is not listed above, please specify here: (7) ____

Display This Question:

If Are you trans or transgender A person whose gender identity differs from the sex they were assign... = Yes

Q42 What is your gender identity? *(Select all that apply)*

▢ Agender A person who does not identify themselves as having a particular gender (1)

▢ Trans Man (2)

▢ Trans Woman (3)

▢ Genderfluid A person who does not identify as having a fixed gender (4)

▢ Non-Binary A person whose gender identity does not fall into the gender binary (i.e. male or female) (5)

▢ Questioning People who feel unsure about their gender identity or sexual orientation may identify with the term 'questioning' (6)

▢ Transfeminine A transgender person who is assigned male at birth and generally identifies with a 'feminine' gender identity greater than a 'masculine' gender identity (25)

▢ Transmasculine A transgender person who is assigned female at birth and generally identifies with a 'masculine' gender identity greater than a 'feminine' gender identity (26)

▢ If the gender identity is not listed above, please specify here: (27) ____

Q43 What is your sexual orientation? *(Select all that apply)*

▢ Asexual A person who does not experience physical attraction and may or may not experience emotional attraction to other people and identifies as asexual (1)

▢ Bisexual A person who is attracted to people of their own gender and other genders and who identifies as bisexual (2)

▢ Demisexual A person who may experience physical attraction when an emotional bond is formed with another person and identifies as demisexual (3)

▢ Gay A man who is attracted to other men and who identifies as gay. The term 'gay' can also be used to describe a person who is attracted to others of the same gender (4)

▢ Lesbian A person who identifies as a woman and who is attracted to other women and who identifies as lesbian (5)

▢ Pansexual A person who is attracted to people regardless of gender (6)

▢ Queer A broader term that includes all sexual orientation and identities within the 2SLGBTQQIA+ communities, including those who do not identify with any other identities listed and feel comfortable with the term 'queer' (7)

▢ Questioning People who feel unsure about their gender identity or sexual orientation may identify with the term 'questioning' (8)

▢ Straight/ Heterosexual A person who is attracted to people of a gender other than their own (9)

▢ If your identity is not listed above, please specify here: (10) ____

End of Block: Gender and Sexual Identity

Start of Block: Understanding your experience at a pharmacy

Q6 **Understanding Your Experience at the Pharmacy**

Please think about the times when you received
healthcare from a pharmacist or went to a pharmacy as you read the statements below. How

strongly do you agree or disagree with these statements?

When I visit my pharmacy,

	Strongly Disagree (61)	Somewhat Disagree (62)	Neither Agree nor Disagree (63)	Somewhat Agree (64)	Strongly Agree (65)	Not Applicable (66)
I feel that the physical environment of the pharmacy is 2SLGBTQQIA+ inclusive. 2SLGBTQQIA+ inclusive spaces may feature progress/pride flag, printed material on 2SLGBTQQIA+ resources, all-gender washrooms, and other displays of inclusivity. (1)	○	○	○	○	○	○
I feel like the pharmacy washrooms are inclusive of all genders. (4)	○	○	○	○	○	○
I feel like there is an adequate selection of shelf-products for my sexual and healthcare needs. (6)	○	○	○	○	○	○
I feel that it is important that there is a 2SLGBTQQIA+ pharmacy staff member whom I can speak to. (3)	○	○	○	○	○	○

Q22 What makes the physical pharmacy environment 2SLGBTQQIA+ inclusive, or not 2SLGBTQQIA+ inclusive?

____

Q23 In general, when I speak to a pharmacist,

	Strongly Disagree (1)	Somewhat Disagree (2)	Neither Agree nor Disagree (3)	Somewhat Agree (4)	Strongly Agree (5)	Not Applicable (6)
I feel respected as a 2SLGBTQQIA+ person. (1)	o	o	o	o	o	o
I feel comfortable sharing my gender and sexual identity if asked. (6)	o	o	o	o	o	o
I feel comfortable correcting my pharmacist about my gender or sexual identity. (8)	o	o	o	o	o	o
I feel that my needs are met as a 2SLGBTQQIA+ person. (7)	o	o	o	o	o	o

Display This Question:

If In general, when I speak to a pharmacist, = I feel that my needs are met as a 2SLGBTQQIA+ person. [Strongly Agree]

Or In general, when I speak to a pharmacist, = I feel that my needs are met as a 2SLGBTQQIA+ person. [Somewhat Agree]

Or In general, when I speak to a pharmacist, = I feel that my needs are met as a 2SLGBTQQIA+ person. [Neither Agree nor Disagree]

Q24 How does the pharmacist meet your needs as a 2SLGBTQQIA+ person?

____

Display This Question:

If In general, when I speak to a pharmacist, = I feel that my needs are met as a 2SLGBTQQIA+ person. [Strongly Disagree]

Or In general, when I speak to a pharmacist, = I feel that my needs are met as a 2SLGBTQQIA+ person. [Somewhat Disagree]

Or In general, when I speak to a pharmacist, = I feel that my needs are met as a 2SLGBTQQIA+ person. [Neither Agree nor Disagree]

Q25 How does the pharmacist **not** meet your needs as a 2SLGBTQQIA+ person?

____

End of Block: Understanding your experience at a pharmacy

Start of Block: Evaluation of Pharmacy Education

Q16 **Training Our Future Pharmacists**

In order to provide inclusive care to 2SLGBTQQIA+ clients, pharmacists must have appropriate training, such as those listed below. Please read the following statements. Drag and arrange statements by order of importance. The **most important** item should be ranked **#1** and the **least important** should be ranked **#8**.

Q17 I want my pharmacist to:
______ Have knowledge about 2SLGBTQQIA+ health topics. 2SLGBTQQIA+ health topics may include but are not limited to providing gender-affirming care, preventing and treating sexually

transmitted diseases including HIV and AIDs, and understanding how to support 2SLGBTQQIA+ folx affected by mental health problems, substance use, and trauma. (1)

______ Address me using pronouns and language I am comfortable with. (10)

______ Create an inclusive physical space that shows me I am accepted for who I am. 2SLGBTQQIA+ inclusive spaces may feature progress/pride flags, printed material on 2SLGBTQQIA+ resources, all-gender washrooms, and other displays of inclusivity. (11)

______ Treat me respectfully without assuming or stereotyping. (12)

______ Listen to me and my experiences as a 2SLGBTQQIA+ person. (13)

______ Know about the history of the 2SLGBTQQIA+ community and the various sexual and gender identities. (14)

______ Recommend local 2SLGBTQQIA+ resources. 2SLGBTQQIA+ resources may include educational information, community support groups, crisis lines, access to 2SLGBTQQIA+ healthcare and services, shelters, and other social resources. (15)

______ Understand how social factors can affect the health of people in my communities. Social factors that affect health may include discrimination, healthcare access, income, education, and social support. (16)

Q34 Are there any topics you feel are missing from the above list? If so, please elaborate below.

____

____

____

____

____

End of Block: Evaluation of Pharmacy Education

Start of Block: Block 6

Q18 Please tell us anything else you would like us to know.

____

____

____

____

____

End of Block: Block 6

Start of Block: Block 7

Q33 Thank you for your time spent taking this survey. If you would like to enter into a random draw for a chance to win a $20 CAD e-gift card, please follow the link below. You will be asked to provide your name and contact information.   Rest assured that this information will not be linked to your answers in this survey in any way, and will only be used to contact you if you win the raffle.

Link: https://ubc.ca1.qualtrics.com/jfe/form/SV_82PHndY3HDssSW2

End of Block: Block 7

Chen LPC, Ng CN, Abdoulrezzak RM, et al. 2SLGBTQ+ patients' experiences in the pharmacy in British Columbia, Canada. *Can Pharm J (Ott)* 2025;158. DOI 10.1177/17151635231360227.
