# Supplementary material for: 2SLGBTQ+ patients’ experiences in the pharmacy in British Columbia, Canada
Source: Can Pharm J (Ott). 2025 Sep 12;158(6):368–77. doi: 10.1177/17151635251360227 (PMC12432006; doi:10.1177/17151635251360227)
Supplement: sj-pdf-2-cph-10.1177_17151635251360227 – Supplemental material for 2SLGBTQ+ patients’ experiences in the pharmacy in British Columbia, Canada [file sj-pdf-2-cph-10.1177_17151635251360227.pdf]

APPENDIX 2

Table A1. Participant-identified themes of inclusive characteristics of the physical pharmacy environment (N = 125).

Common Theme	Percent (%)	Count (n)	Example (s)
2SLGBTQ+ signage, stickers, or pins	20.0%	25	"If there is a progress flag or if the people are wearing pronoun pins/flag pins, I feel immediately at ease."<br><br>"Even something as simple as advertising. Posters on the wall that include queer people or couples. Pamphlets for more queer-specific services/information. … A rainbow sticker somewhere … letting people know they can comfortably share information about their identity (or their bodies), or feel comfortable asking questions, especially if there's a certain pharmacist they can go to."
Open-minded, friendly, respectful, or 2SLGBTQ+ personnel	19.2%	24	"I think a pharmacy environment is a neutral space that isn't physically inclusive or exclusive. The only thing I can think of as necessary for inclusivity in a pharmacy is the workers' actions and words which I assume isn't apart [sic] of the physical environment."
Pharmacists with competency training or knowledgeable staff	14.4%	18	"Just respect for queer patients is inclusive. Non-judgment pharmacy staff is good and being educated on LGBTQ health would be great."
Use of inclusive language (e.g., asking and using chosen names and pronouns)	12.0%	15	"Initiating the question about pronouns [and] names [is important] … once in a blue moon the reception gets it and we [are] … happy and seen."

Space conducive to autonomy and privacy	9.6%	12	“Privacy is a good part of being inclusive; many pharmacies just don't have the physical infrastructure to maximize privacy, which may make some feel uncomfortable.”
2SLGBTQ+ specific products	6.4%	8	“A great inclusive action to take is carrying products that specifically cater to queer and trans needs, such as information/safety booklets and products for binding and tucking. Carrying products that are not divided by "mens" and "womens" colours or smells is great too.”
Use of inclusive forms and documentation	4.8%	6	“adding a preferred name option to customer databases, using the pronoun "they" as a defualt [sic], or ideally referring to customers by name rather than pronouns.”<br><br>“when they have the option of entering your preferred name in the system and actually use it to talk to you.”

2SLGBTQ+, Two-Spirit, lesbian, gay, bisexual, transgender, queer, intersex, and additional people who identify as part of sexual and gender diverse communities.

Table A2: Participant-identified themes of non-inclusive characteristics of the physical pharmacy environment (N = 125).

Theme	Percent (%)	Count (n)	Example(s)
Non-private, non-inclusive environment	11.2%	14	"There are all those signs, cardboard selves, etc., of MEN's multi vitamins or WOMEN' whatever. Just an extra reminder of what I'm not when picking up my hormones."<br><br>"What makes [the pharmacy environment] not inclusive: using terms like "feminine hygiene products", lack of access to a wheelchair accessible/gender neutral restroom …"<br><br>"…lack of privacy during consultation with pharmacists [is part of what makes pharmacies non-inclusive]."
Use of non-inclusive language	10.4%	13	"Pharmacies are not very respecting of chosen names in my experience, and sometimes you get weird looks or glares from the employees."
Untrained personnel or lack of knowledge	9.6%	12	"Often when asking for needles for hormones I am questioned by pharmacy workers, and sometimes the needles/syringes for the injections I need is not supplied, therefore I have to travel farther for injection supplies, which is not accessible for many people."<br><br>"lack of knowledge by staff regarding how medications/treatments/other effect [sic] others differently based on their gender identity and sexual orientation history [makes a pharmacy non-inclusive]."
Non-inclusive products or lack of products	9.6%	12	"[Non-inclusive pharmacies have a] lack of safer sex products, [and have] condoms lube only available in "family planning" section."

Personnel; non-inclusive, closed-minded, questioning	8.8%	11	"Being misgendered and deadnamed; staff appearing fearful of interacting with me based on body/facial language; staff lying or intentionally creating barriers to getting needles for HRT."
No inclusive signs/ stickers/ pins, or cisheteronormative signage	6.4%	8	"There are no indications of any acknowledgment of the 2SLGBTQIA+ community. A lot of products and pharmacy photos/promotions have images of cisgender and heterosexual couples/families and there are not visual indications that the pharmacists acknowledge queer patients like those "safe space" stickers (which I think are usually a sign that at least they are trying to look queer-friendly)."
Non-inclusive forms	1.6%	2	"use of non-inclusive language by staff (pharmacists) and in medical forms."

HRT, hormone replacement therapy; 2SLGBTQIA+, Two-Spirit, Lesbian, Gay, Bisexual, Transgender, Queer, Intersex, Asexual, and other identities within the broader community of sexual and gender diverse people.

Table A3. Participant-identified themes of how a pharmacist meets participants' needs as a 2SLGBTQ+ person. (N = 81)

Common theme	Percent (%)	Count (n)	Example(s)
Clinical competence	43.2%	35	"My pharmacist worked w[ith] me through medical transition and was supportive, kind, and respectful."<br><br>"The pharmacist that has been the most helpful has been able to answer my questions on how to inject hormones, the side effects, and provide an overall comforting environment."
Open-minded, respectful demeanour	40.7%	33	"[The pharmacist's] dedication to inclusivity is clear in their demeanour … They do not make it weird everything is just 'normal' … I feel comfortable and not judged for the medication I have to fill and the advice they provide is gender-neutral. For example, I have to purchase birth control but they do not make me feel like this is exclusively a 'female' or woman's medication."
Non-assuming and non-invasive care	12.3%	10	"[Inclusive pharmacists] fill my prescriptions right and [do] not question what they are seeing (e.g., incongruence between prescription and gender marker/names in different places/paper and physical appearances)."
Using correct pronouns and gender-neutral language	9.9%	8	"I like when [pharmacists'] name tags have pronouns, and when they use neutral language. In general, feeling respected as a person makes me feel like being queer won't affect my care."
Not applicable: participant does not have 2SLGBTQ+ needs	7.4%	6	"I don't have a lot of queer needs at the pharmacy."<br><br>"Just meeting basic human needs. Most of the time people wouldn't assume that I'm a part of the LGBTQ."
Informed about 2SLGBTQ+ experience	6.2%	5	"[Pharmacists can meet my needs] by being helpful and informed about trans people without having to question me or my prescription. Helpful when I have questions regarding needles or ordering a larger

			supply of them and not question why I need so many, my prescription should be enough info as to why."
Efficient service	2.5%	2	"They give me my medications with as little interaction as possible."
2SLGBTQ+ advocacy/ community involvement	1.2%	1	"… I use a pharmacy that has donated to LGBT organizations locally."

2SLGBTQ+, Two-Spirit, lesbian, gay, bisexual, transgender, queer, intersex, and additional people who identify as part of sexual and gender diverse communities.

Table A4. Participant-identified themes of how a pharmacist does not meet participants' needs as a 2SLGBTQ+ person (N = 52).

Common theme	Percent (%)	Count (n)	Example(s)
Lack of clinical competency	46.2%	24	"When I went on testosterone gel, I wanted to ask the pharmacist about any side effects I might experience. At first, all they said was 'masculinizing effects', and when I said that I was trans so that was the point, he looked physically uncomfortable and basically just said there was nothing else and walked away."
Intrusive, heteronormative assumptions or questioning	28.8%	15	"Often I am asked about my husband when it comes to extended health benefits. I do not have a husband; I have a wife."
Misgendering or deadnaming	25.0%	13	"They are unable to update my name in their system despite having a legal name change 6+ years ago and all my identity documents being changed. This results in my former name being called when I am called to pick up prescriptions, etc."
Disrespectful personnel or denial of service	19.2%	10	"I found that some pharmacies feel entitled to deny coverage for testosterone for people even with special approval/physical doctor permission. If someone can't put their job before their views they should not be in medicine or social services."
Lack of 2SLGBTQ+ services, supplies, or information	9.6%	5	"My pharmacy does not offer any information on health for trans people who bind or use hormones."
Lack of privacy	9.6%	5	"Back when I first started HRT, going to pick up my hormones was a terrifying, stressful process. There's no privacy, anyone could hear what I was getting. They could see they [sic] look on the pharmacist's face. They could hear the 'are you sure this is the correct medication? You aren't picking up for someone else?'"

Table A5: Pairwise comparisons of the top 3 most highly ranked pharmacist competencies.

	"Respect"	"Knowledge"	"Language"	Sum
Number of responses where "Respect" ranked higher than…	-	88	100	188
Number of responses where "Knowledge" ranked higher than…	72	-	94	166
Number of responses where "Language" ranked higher than…	60	66	-	126

Chen LPC, Ng CN, Abdoulrezzak RM, et al. 2SLGBTQ+ patients' experiences in the pharmacy in British Columbia, Canada. *Can Pharm J (Ott)* 2025;158. DOI 10.1177/17151635231360227.
