# Supplementary material for: 2SLGBTQ+ patients’ experiences in the pharmacy in British Columbia, Canada
Source: Can Pharm J (Ott). 2025 Sep 12;158(6):368–77. doi: 10.1177/17151635251360227 (PMC12432006; doi:10.1177/17151635251360227)
Supplement: sj-pdf-3-cph-10.1177_17151635251360227 – Supplemental material for 2SLGBTQ+ patients’ experiences in the pharmacy in British Columbia, Canada [file sj-pdf-3-cph-10.1177_17151635251360227.pdf]

APPENDIX 3

Figure A1: Histogram distributions of ranks assigned to each pharmacist competency. A rank of "1" indicates the participant thought that item was the most important, and "8" indicated least important.

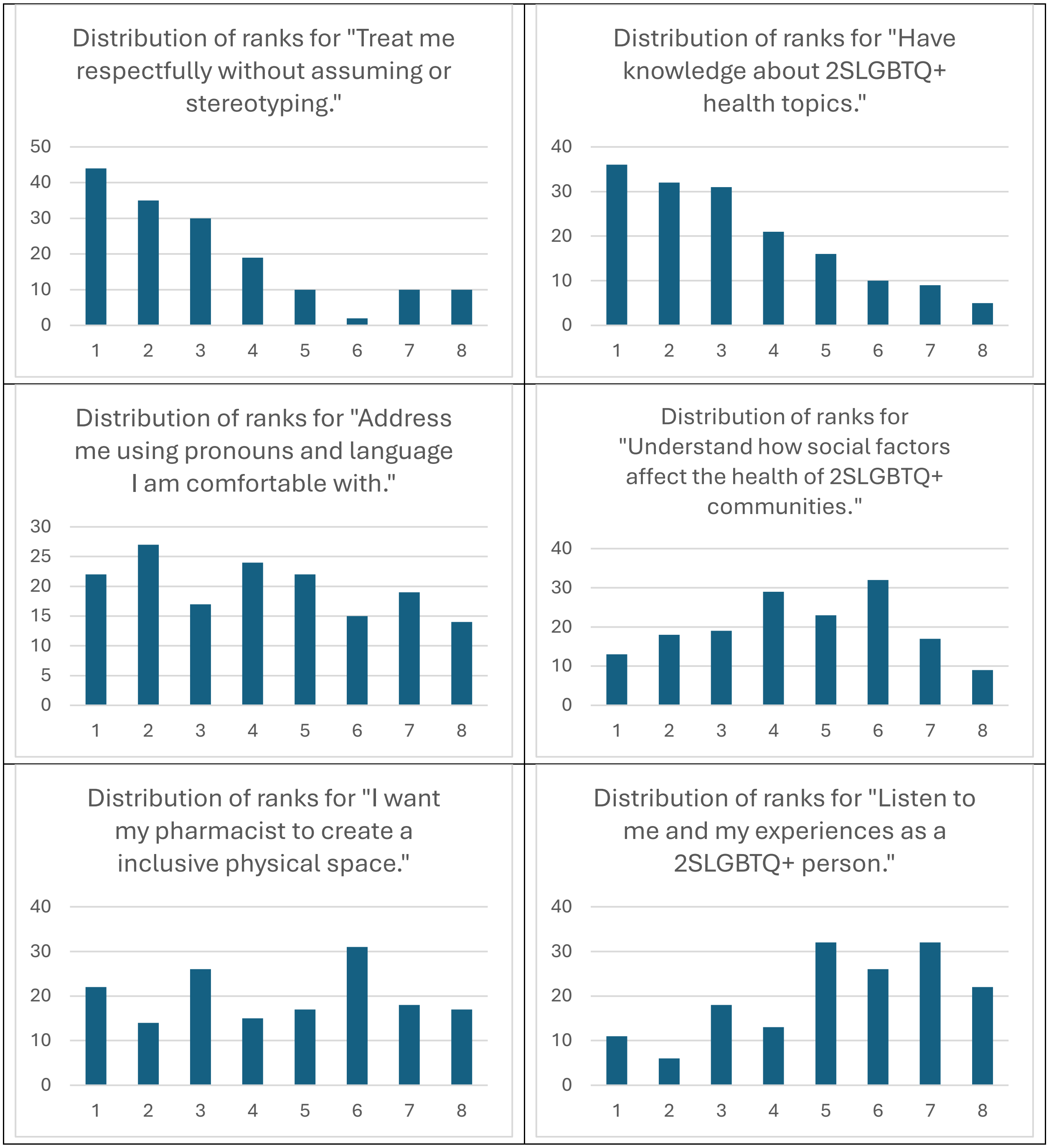

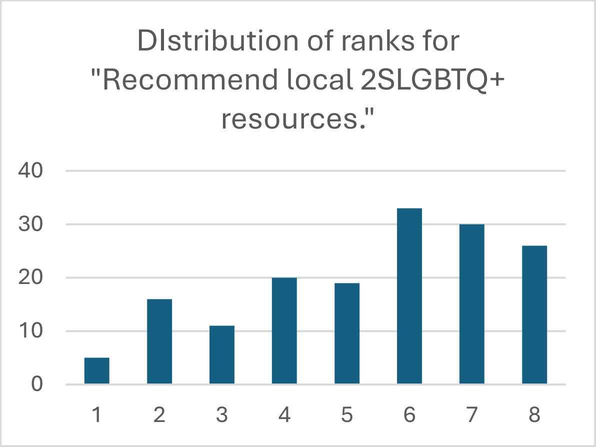

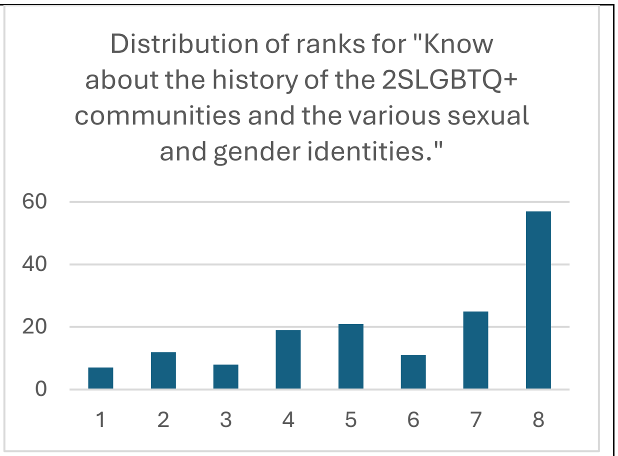

2SLGBTQ+, Two-Spirit, lesbian, gay, bisexual, transgender, queer, intersex, and additional people who identify as part of sexual and gender diverse communities.

Chen LPC, Ng CN, Abdoulrezzak RM, et al. 2SLGBTQ+ patients' experiences in the pharmacy in British Columbia, Canada. *Can Pharm J (Ott)* 2025;158. DOI 10.1177/17151635231360227.
